# Metals in Urine and Peripheral Arterial Disease

**DOI:** 10.1289/ehp.7329

**Published:** 2004-11-22

**Authors:** Ana Navas-Acien, Ellen K. Silbergeld, A. Richey Sharrett, Emma Calderon-Aranda, Elizabeth Selvin, Eliseo Guallar

**Affiliations:** ^1^Department of Epidemiology, Johns Hopkins University Bloomberg School of Public Health, Baltimore, Maryland, USA; ^2^Welch Center for Prevention, Epidemiology, and Clinical Research, Johns Hopkins Medical Institutions, Baltimore, Maryland, USA; ^3^Johns Hopkins Center for Excellence in Environmental Public Health Tracking, Baltimore, Maryland, USA; ^4^Department of Environmental Health Sciences, Johns Hopkins University Bloomberg School of Public Health, Baltimore, Maryland, USA; ^5^Sección de Toxicología, Cinvestav, México

**Keywords:** antimony, atherosclerosis, cadmium, lead, metals, peripheral arterial disease, tungsten

## Abstract

Exposure to metals may promote atherosclerosis. Blood cadmium and lead were associated with peripheral arterial disease (PAD) in the 1999–2000 National Health and Nutrition Examination Survey (NHANES). In the present study we evaluated the association between urinary levels of cadmium, lead, barium, cobalt, cesium, molybdenum, antimony, thallium, and tungsten with PAD in a cross-sectional analysis of 790 participants ≥40 years of age in NHANES 1999–2000. PAD was defined as a blood pressure ankle brachial index < 0.9 in at least one leg. Metals were measured in casual (spot) urine specimens by inductively coupled plasma–mass spectrometry. After multivariable adjustment, subjects with PAD had 36% higher levels of cadmium in urine and 49% higher levels of tungsten compared with noncases. The adjusted odds ratio for PAD comparing the 75th to the 25th percentile of the cadmium distribution was 3.05 [95% confidence interval (CI), 0.97 to 9.58]; that for tungsten was 2.25 (95% CI, 0.97 to 5.24). PAD risk increased sharply at low levels of antimony and remained elevated beyond 0.1 μg/L. PAD was not associated with other metals. In conclusion, urinary cadmium, tungsten, and possibly antimony were associated with PAD in a representative sample of the U.S. population. For cadmium, these results strengthen previous findings using blood cadmium as a biomarker, and they support its role in atherosclerosis. For tungsten and antimony, these results need to be interpreted cautiously in the context of an exploratory analysis but deserve further study. Other metals in urine were not associated with PAD at the levels found in the general population.

Peripheral arterial disease (PAD) is a consequence of atherosclerotic occlusion of blood flow in the muscular arteries of the lower extremities. Because traditional risk factors for atherosclerosis, such as age, smoking, hypertension, and diabetes, do not completely explain the distribution of PAD in the population, there is considerable interest in identifying novel, nontraditional risk factors. Certain metals may promote atherosclerosis by increasing oxidative stress [e.g., by catalyzing the production of reactive oxygen species or inhibiting their degradation (Stohs and Bagchi 1995)] or by affecting other cardiovascular risk factors [e.g., by increasing blood pressure levels (Nawrot et al. 2002; Revis et al. 1981)]. Arsenic, for instance, has been associated with PAD and with other forms of atherosclerosis in populations exposed to arsenic in drinking water (Carter et al. 2003; National Research Council 1999). However, evidence for an association between most metals and PAD is limited.

Blood cadmium and lead, at levels well below current safety standards, were associated with an increased prevalence of PAD in the 1999–2000 National Health and Nutrition Examination Survey (NHANES) (Navas-Acien et al. 2004). Cadmium and lead have been associated with other cardiovascular end points, such as myocardial infarction and stroke, in some studies (Lustberg and Silbergeld 2002; Ponteva et al. 1979) but not all (Pocock et al. 1988; Staessen et al. 1996). For several other metals, including barium, cobalt, molybdenum, antimony, and thallium, data on their role in human atherosclerosis are sparse (Brenniman et al. 1979; Guo et al. 1992; Heim et al. 2002; Schnorr et al. 1995). There are no epidemiologic data on the potential cardiovascular effects of cesium or tungsten.

The objective of this study was to evaluate the association of metal levels in urine with the prevalence of PAD in NHANES 1999–2000, a representative sample of the civilian, noninstitutionalized U.S. population. In epidemiologic studies, PAD is usually defined by the presence of a low (< 0.9) blood pressure ankle-brachial index (ABI)—that is, when the systolic blood pressure measured at the ankle is < 90% of the systolic blood pressure measured at the arm (Selvin and Erlinger 2004). We hypothesized that cadmium and lead would be positively associated with PAD, as determined by an ABI < 0.9. In addition, we explored the association of PAD with urinary barium, cobalt, cesium, molybdenum, antimony, thallium, and tungsten, without prior hypotheses. These metals were selected for study in NHANES 1999–2000 by the National Center for Health Statistics (NCHS). The widespread exposure to these metals in the general population [National Center for Environmental Health (NCEH) 2003] and the lack of relevant population data for most of them underscore the public health relevance of this study.

## Materials and Methods

### Study population.

The NCHS has conducted a series of cross-sectional health and nutrition surveys beginning in the 1960s. These surveys have used a stratified, multistage probability cluster design to provide data representing the noninstitutionalized U.S. population (NCHS 2004a). In 1999, NHANES became a continuous survey and also began collecting information on ABI in men and women ≥40 years of age (NCHS 2004b).

Among the 9,965 participants in NHANES 1999–2000, one-third of the study population ≥6 years of age were randomly selected to obtain urinary measurements of metals (*n* = 2,465). NHANES 1999–2000 included a detailed lower-extremity examination that involved ABI measurements in subjects ≥40 years of age with no bilateral amputations and weighing < 400 lb (*n* = 2,875, of whom 796 had a determination of urinary metals). From these 796 participants, we excluded one participant with an ABI value > 1.5 [usually related to noncompressible vessels in the legs (Newman et al. 1993)] and five participants with no information on smoking status or urine levels of creatinine, for a final sample size of 790 participants. A few remaining participants had missing data for some of the metals (sample size available for each metal shown in Table 1). The response rate for the household interview and the physical exam components of NHANES 1999–2000 were 82 and 76%, respectively (NCHS 2004b).

### PAD.

A specific protocol was used to measure ABI in NHANES 1999–2000 (NCHS 2004b). The measurements of blood pressure used for ABI were different from the measurements of blood pressure used to define hypertension. Supine systolic blood pressure was measured on the right arm (brachial artery) and both ankles (posterior tibial arteries) using a Doppler device, the Parks Mini-Lab IV, model 3100 (Parks Medical Electronics, Aloha, OR, USA). If the participant had a condition that would interfere with blood pressure reading in the right arm, the left arm was used. Systolic blood pressure was measured twice at each site for participants 40–59 years of age and once at each site for participants ≥60 years of age. The measurements for left and right ABI were obtained by dividing the mean ankle systolic blood pressure in each side by the mean brachial systolic blood pressure. PAD was defined as an ABI value < 0.90 in at least one leg (Newman et al. 1993).

### Urinary metals.

A casual (or spot) urine specimen was collected from the participants after confirmation of no background contamination in collection materials (NCHS 2004b). Urinary levels of cadmium, lead, barium, beryllium, cobalt, cesium, molybdenum, platinum, antimony, thallium, and tungsten were measured at the Environmental Health Sciences Laboratory of the Centers for Disease Control and Prevention (CDC) and the National Center for Environmental Health (NCEH) by inductively coupled plasma–mass spectrometry (PerkinElmer/SCIEX model 500; PerkinElmer, Shelton, CT, USA) using a multielement analytical technique following published protocols (NCHS 2004c; Paschal et al. 1998). Cadmium levels in urine were corrected for interference from molybdenum oxide (NCHS 2004c). Urine Standard Reference Material 2670 from the National Institute of Standards and Technology (Gaithersburg, MD, USA) was used for external calibration and spiked pools prepared at the laboratory were used for internal quality control. Quality control samples included both bench and blind samples. The ranges for the interassay coefficients of variation for each metal are shown in Table 1. The limits of detection varied by metal, from 0.01 μg/L for thallium to 0.85 μg/L for molybdenum (Table 1). The levels of beryllium and platinum were below the limit of detection in > 98% of participants and were not considered further. For the other metals, the percentage of subjects with levels below the limit of detection ranged from 0.5% for cesium to 30% for tungsten (Table 1). A level equal to the limit of detection divided by the square root of 2 was imputed for those subjects with levels below the limits of detection.

### Other variables.

Information on age, sex, race/ethnicity, education, and smoking was based on self-report. Urinary creatinine was determined using an enzymatic reaction measured with a CX3 analyzer (NCHS 2004b).

### Statistical analysis.

All statistical analyses were performed using SUDAAN software (Research Triangle Institute, Research Triangle Park, NC, USA) to account for the complex sampling design and weights in the NHANES 1999–2000 metal subsample. The jackknife “leave-one-out” method was used to obtain appropriate standard errors of all estimates.

Urinary metal levels were right skewed and were log-transformed to improve normality. For each metal, the ratio of the geometric mean urinary level and its 95% confidence interval (CI) comparing subjects with PAD versus subjects without PAD were estimated using linear regression models on log-transformed metal levels. For risk analyses, logistic regression was used to obtain the adjusted odds ratios and 95% CIs of PAD comparing the 75th with the 25th percentile of the distribution of each metal assuming a log-linear relationship between urinary metals and PAD. We also used restricted cubic spline transformations to assess nonlinear relationships. All models were adjusted for age, sex, race, and education level. In addition, we evaluated the impact of further adjusting for smoking status, a source of cadmium, lead, and cobalt (International Agency for Research on Cancer 1986). Metal levels were based in one casual urine sample and thus depend on urine dilution. Urinary metal levels were adjusted for urinary creatinine to correct for differences in urine dilution in spot urine samples, but because of the limitations of creatinine concentration to adjust for urine dilution (Ikeda et al. 2003), we present results both with and without creatinine adjustment. We also repeated the analyses excluding subjects fasting < 8 hr to evaluate the impact of postprandial or fasting state and rapid renal clearance of some metals. No substantial changes were observed (data not shown). We also assessed confounding by other cardiovascular risk factors, including hypertension, hypercholesterolemia, diabetes, estimated glomerular filtration rate (Coresh et al. 2002), and C-reactive protein, by adding them one at a time to the multivariable models. No noticeable changes were observed (data not shown), and because of the limited sample size, these additional variables were not included in the final models.

## Results

### Metal levels in urine.

**Figure 1:**
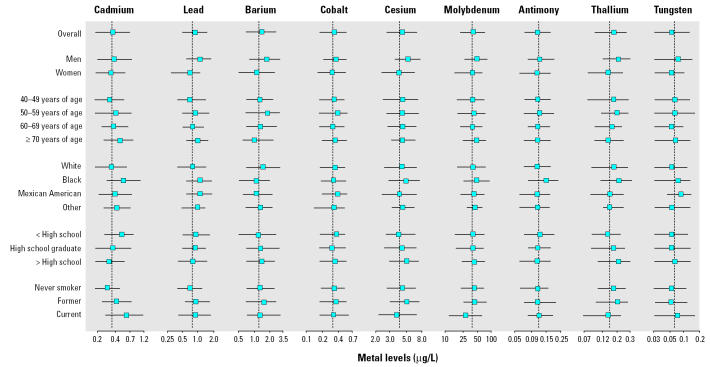
Metal levels in urine (μg/L) by participant characteristics. Horizontal lines, interquartile ranges; squares, medians; dotted vertical line, the geometric mean for the overall study sample.

Table 1 describes urinary metal levels in the study sample. The geometric means were lowest for tungsten, 0.07 μg/L, and highest for molybdenum, 37.7 μg/L. The median level and the interquartile range for each metal by participant characteristics are shown in Figure 1. Men had higher levels than women for most metals, and cadmium and lead tended to increase with age. Compared with whites, blacks and Mexican Americans had higher levels of cadmium, lead, and tungsten, and blacks had higher levels of cesium, molybdenum, antimony, and thallium. Current smokers had higher levels of cadmium, lead, antimony, and tungsten compared with never smokers.

### Metals and PAD.

**Figure 2:**
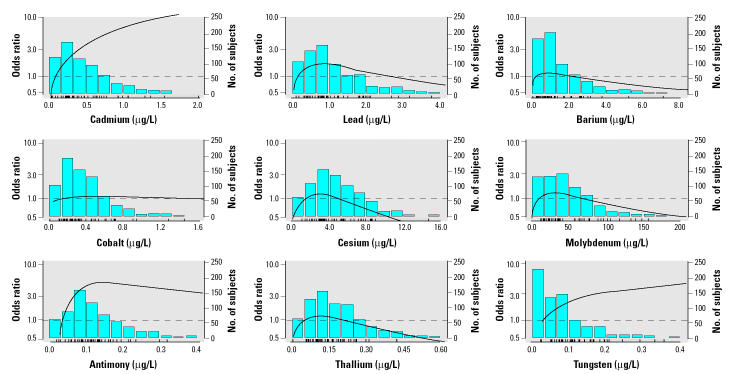
Odds ratios of PAD by metal levels in urine. The curves are odds ratios adjusted for age, sex, race, education, smoking, and urinary creatinine based on restricted cubic spline transformations. The reference value (odds ratio = 1) was set at the 10th percentile of the distribution for each metal. The bar histograms represent the frequency distribution of each metal in the study sample. The tick marks at the bottom of the histogram represent the metal level of the cases of PAD.

The overall prevalence of PAD in the study sample was 5.4% (54 cases). After adjusting for demographic variables, smoking status, and creatinine in urine, subjects with PAD had 36% (95% CI, 1 to 83) higher mean levels of cadmium in urine compared with subjects without PAD (Table 2). Levels of lead, barium, cobalt, cesium, molybdenum, antimony, and thallium were similar in subjects with and without PAD. Subjects with PAD had 49% (95% CI, −10 to 249) higher mean levels of tungsten. The association of cadmium and tungsten with PAD was also evident in logistic models (Table 3, Figure 2). The odds ratio for PAD comparing the 75th with the 25th percentile of the cadmium distribution was 3.05 (95% CI, 0.97 to 9.58). The corresponding odds ratio for tungsten was 2.25 (95% CI, 0.97 to 5.24). For antimony, no association was apparent in linear models (Tables 2 and 3), but nonlinear models showed an increase in the prevalence of PAD at very low levels and a plateau of risk > 0.1 μg/L (Figure 2).

## Discussion

### Cadmium.

Cadmium levels in urine were 36% higher in subjects with PAD than in those without PAD. This association was similar but actually stronger than the previously reported association between blood cadmium and PAD in NHANES 1999–2000, where blood cadmium was 16% higher in PAD cases than in noncases (Navas-Acien et al. 2004). The stronger association for urinary compared with blood cadmium probably reflects the fact that urinary cadmium is a more reliable biomarker of chronic cadmium exposure than blood cadmium (ATSDR 1999a; Trzcinka-Ochocka et al. 2004). The association between cadmium levels and PAD was not explained by smoking status, and adjusting for smoking decreased only slightly the magnitude of the association. In our study, an increased prevalence of PAD was associated with low levels of urinary cadmium, below the levels reported in workers exposed to cadmium occupationally (Olsson et al. 2002). For example, only two subjects had cadmium levels > 3 μg/g of creatinine, the Occupational Safety and Health Administration safety standard for cadmium in urine (Occupational Safety and Health Administration 2003). In the NHANES population, because occupational exposure is expected to be relatively rare, it is likely that exposure to cadmium occurred mainly through cigarette smoking, inhalation of airborne cadmium in ambient air (usually higher near coal-fired power plants and municipal waste incinerators), or consumption of some foods (highest levels in shellfish, liver, and kidney meats) (ATSDR 1999b).

Several mechanisms may explain an increased risk of atherosclerosis with cadmium, including the catalysis of reactive oxygen species (Stohs and Bagchi 1995; Vaziri et al. 2001), the promotion of lipid peroxidation (Ding et al. 2000; Yiin et al. 1999), the depletion of glutathione and protein-bound sulf-hydryl groups (Stohs and Bagchi 1995), the production of inflammatory cytokines (Heo et al. 1996), and the down-regulation of nitric oxide production (Demontis et al. 1998; Vaziri et al. 2001). Cadmium has also induced atherosclerosis and hypertension in some animal models *in vivo* (Revis et al. 1981). However, these mechanistic studies were typically conducted at considerably higher exposures than those corresponding to the urinary concentrations observed in the present study, and hence their relevance to human atherogenesis and to PAD is uncertain.

Epidemiologic studies of cadmium and cardiovascular disease are also limited. Ecologic studies have found associations of cardiovascular mortality rates with cadmium levels in air (Carroll 1966) and in soil and water (Houtman 1993). Two small case–control studies found higher blood cadmium in subjects with myocardial infarction than in controls (Adamska-Dyniewska et al. 1982; Ponteva et al. 1979), but a cross-sectional study in Belgium found no association between blood cadmium and the prevalence of cardiovascular disease (Staessen et al. 1996). Finally, several autopsy studies have found associations between tissue cadmium levels and atherosclerotic lesions (Aalbers and Houtman 1985; Voors et al. 1982). Additional studies, particularly with a prospective design, are needed to confirm the role of cadmium in the pathogenesis of PAD and to determine its role in other cardiovascular end points.

### Lead.

Lead in urine was not associated with PAD in this study. This result is in contrast to the association observed between blood lead and PAD in NHANES 1999–2000, where blood lead levels were 14% higher in cases of PAD than in noncases (Navas-Acien et al. 2004). This discrepancy could be related to the fact that urinary lead levels in this population were low and may have considerable fluctuations when evaluated in spot urine samples. Under these conditions, urinary lead is considered a less reliable biomarker of exposure than blood lead (ATSDR 1999b). Indeed, previous cohort studies of lead and cardiovascular disease have used blood lead as the biomarker of exposure. Blood lead was positively associated with cardiovascular mortality in NHANES II (Lustberg and Silbergeld 2002) and with coronary heart disease incidence in Denmark (Moller and Kristensen 1992), although an earlier study in British men did not show an association between blood lead and cardiovascular disease incidence (Pocock et al. 1988). Several mechanisms support a role for lead in atherosclerosis. Lead increases blood pressure (Nawrot et al. 2002; Revis et al. 1981), and experimental studies show that lead promotes oxidative stress (Stohs and Bagchi 1995), stimulates inflammation (Heo et al. 1996), and induces endothelial damage (Vaziri et al. 2001). The role of lead in the development of atherosclerosis, however, needs to be further investigated in mechanistic studies at low levels of exposure and in prospective studies in humans using appropriate biomarkers of chronic exposure.

### Tungsten.

Tungsten levels in urine were 49% higher in subjects with PAD than in those without PAD. This analysis is among the first to examine the role of tungsten in any health status indicator, and it needs to be interpreted cautiously in the context of multiple comparisons. Little is known regarding tungsten toxicity and carcinogenicity (ATSDR 2003), and there are insufficient data, either from animal or human studies, on its cardiovascular effects (Lagarde and Leroy 2002). It is known, however, that tungsten is thrombogenic and proinflammatory (Byrne et al. 1997). In fact, these properties have motivated the clinical use of tungsten coils for the occlusion of intracranial aneurysms, varicocele veins, and other abnormal vascular connections (Butler et al. 2000; Peuster et al. 2002). In addition, tungsten may interfere with the biologic effect of molybdenum, an essential trace element that acts as cofactor in several proteins (Johnson et al. 1974; Nell et al. 1980). For instance, tungsten inhibits xanthine oxidase (Johnson et al. 1974), an antioxidant molybdoenzyme with a role in endothelial dysfunction (Berry and Hare 2004) and in the maintenance of the vessel wall integrity.

Sources of tungsten exposure include occupational use of tungsten inert gas, welding, and sometimes drinking water. The amounts of tungsten in foods and in ambient air are generally unknown (ATSDR 2003). Urban settings tend to have higher levels of tungsten in the air because tungsten can be released from industrial sources and waste incinerators (tungsten filaments are used in incandescent light bulbs) (ATSDR 2003). In response to the lack of knowledge on the health effects of tungsten exposure coupled with evidence of widespread human exposure, the CDC/NCEH nominated tungsten in 2002 to the National Toxicology Program as a priority candidate for toxicologic assessment (U.S. Department of Health and Human Services 2003). Our findings suggest that these studies should also include the evaluation of the relationship of tungsten to cardiovascular end points and its potential mechanisms of action.

### Antimony.

For antimony, there was an increase in the prevalence of PAD in subjects with low levels compared with those at the limit of detection, and the risk remained increased > 0.1 μg/L. The general population is exposed to antimony in food, in drinking water, and in ambient air (ATSDR 1992a). At levels found in antimony smelting plants, antimony has been related to pneumoconiosis and dermatitis (McCallum 1989). Although antimony is a well-known toxic metal at high doses, there is only one available study of chronic antimony exposure and cardiovascular end points in humans (Schnorr et al. 1995). This study compared coronary mortality in Mexican-American antimony smelter workers with other groups of workers, but the findings were inconclusive. Interestingly, antimony shares similar chemical and toxicologic properties with arsenic (Gebel 1997), and they are frequent coexposures (Gebel et al. 1998). Arsenic exposure has been associated with PAD, and “blackfoot disease” is a classic sign of high arsenic exposure (Carter et al. 2003; National Research Council 1999). Unfortunately, arsenic levels were not measured in NHANES 1999–2000, and we cannot discard the possibility that the observed association between PAD and antimony was due to arsenic coexposure. Further studies are required on antimony and cardiovascular end points.

### Other metals.

Barium, cobalt, cesium, molybdenum, and thallium in urine were not associated with PAD in this representative sample of the U.S. population. Data on the possible role of these metals in atherosclerosis are scarce. Ecologic studies have found positive associations with cardiovascular disease for barium (Brenniman et al. 1979) and thallium (Heim et al. 2002) and a negative association for molybdenum (Guo et al. 1992). Thallium is a poison at high dose (ATSDR 1992b). At low dose, thallium is used in cardiac imaging and thought to be relatively safe (Ranhosky and Kempthorne-Rawson 1990). Cobalt exposure in the hard metal industry results in cardiomiopathy (Seghizzi et al. 1994), but no information is available on its atherogenicity.

### Limitations and strengths.

Our analysis was limited by a relatively small number of cases, because NHANES 1999–2000 measured metals in urine in just one-third of survey participants. This sample size limited the investigation of interactions (e.g., lead and cadmium, or tungsten and molybdenum) and makes it possible that we missed weak associations between some metals and PAD. At the same time, our prior hypotheses regarding the associations of cadmium and lead with PAD were specific, but for the other metals our analyses were exploratory and need to be confirmed in future studies.

Other limitations include the cross-sectional design of the study, the possibility of residual confounding by socioeconomic status or urbanization, the use of a single measurement of urinary metals, and the lack of 24-hr urine collection to better account for short-term variability in metal excretion and urine dilution. To correct for urine dilution, we adjusted for urinary creatinine. However, creatinine in urine is a marker of both urine dilution and creatinine production, and it is associated with factors that affect production such as sex, age, race, and muscle mass. The adequacy of correcting for creatinine has been questioned (Ikeda et al. 2003), and in fact the Second National Report on Human Exposure to Environmental Chemicals (NCEH 2003), based on NHANES data, presented metal concentrations in urine both ways, with and without correction for urinary creatinine. In our data, the findings using models with and without adjustment for creatinine were similar and do not affect the conclusions.

Despite these limitations, this study is the first systematic investigation of a panel of metals in urine with PAD. The use of a representative sample of the U.S. population, rigorous laboratory methods with extensive quality control, and the availability of a standardized procedure to measure ABI add to the strengths of this study.

## Conclusions

Urinary cadmium was strongly associated with PAD in a representative sample of the general U.S. population. This finding strengthens similar results from a previous study in the same population using blood cadmium as a biomarker of exposure (Navas-Acien et al. 2004) and further supports a possible role for cadmium in atherosclerosis. Tungsten was associated with PAD throughout the range of exposure, whereas antimony showed a positive association at low levels that reached a plateau > 0.1 μg/L. These associations need to be interpreted cautiously and require confirmation in future epidemiologic studies and supporting evidence from mechanistic research. Finally, no association between PAD and other metals in urine (lead, barium, cobalt, cesium, molybdenum, and thallium) was evident at the levels found in the general population. The observation of widespread exposure, particularly to poorly studied metals such as tungsten and antimony, supports further research on the role of metals and PAD.

## Figures and Tables

**Table 1:** Metal levels (μg/L) in urine.

	Cadmium	Lead	Barium	Cobalt	Cesium	Molybdenum	Antimony	Thallium	Tungsten
Sample size	728	790	704	790	790	728	725	776	751
Geometric mean	0.36	0.79	1.28	0.31	4.09	37.7	0.11	0.16	0.07
Percentile									
10th	0.10	0.20	0.30	0.10	1.50	11.0	< LOD	0.06	< LOD
25th	0.19	0.50	0.70	0.18	2.80	21.2	0.07	0.10	< LOD
50th	0.36	0.90	1.40	0.33	4.50	41.2	0.11	0.18	0.06
75th	0.67	1.50	2.60	0.53	6.80	71.3	0.17	0.26	0.13
90th	1.16	2.30	4.70	0.81	9.40	126.1	0.29	0.38	0.26
Maximum	12.8	31.5	42.2	556.6	67.4	683.5	5.70	0.86	4.74
LOD	0.06	0.10	0.08	0.07	0.10	0.85	0.04	0.01	0.04
Percent < LOD	1.5	1.8	3.3	3.4	0.5	0.6	9.0	1.3	30.0
Range of CV (%)	1.2–4.7	1.0–5.3	1.4–6.2	1.8–6.0	1.5–9.2	0.6–5.6	1.3–4.8	1.2–8.5	1.1–2.8

Abbreviations: CV, coefficient of variation; LOD, limit of detection.

**Table 2:** Ratios (95% CIs) of the geometric means of metal levels in urine (μg/L) in PAD cases versus noncases.

	Cases	Noncases	Model 1*^a^*	Model 2*^b^*	Model 3*^c^*
Cadmium	49	679	1.81 (1.24–2.62)	1.62 (1.19–2.21)	1.36 (1.01–1.83)
Lead	54	736	1.09 (0.86–1.37)	1.08 (0.85–1.38)	0.92 (0.74–1.15)
Barium	45	659	0.99 (0.67–1.47)	0.99 (0.68–1.45)	0.82 (0.60–1.11)
Cobalt	54	736	1.13 (0.80–1.59)	1.13 (0.82–1.57)	0.98 (0.69–1.40)
Cesium	54	736	1.05 (0.83–1.32)	1.12 (0.89–1.42)	0.96 (0.79–1.16)
Molybdenum	49	679	0.97 (0.66–1.42)	1.08 (0.74–1.57)	0.91 (0.72–1.15)
Antimony	49	676	1.18 (0.92–1.51)	1.17 (0.92–1.50)	1.03 (0.87–1.22)
Thallium	54	722	0.97 (0.71–1.34)	1.08 (0.78–1.48)	0.94 (0.74–1.19)
Tungsten	51	700	1.75 (0.98–3.10)	1.67 (0.96–2.89)	1.49 (0.90–2.49)

a Adjusted by age, sex, race, and education.

b Further adjusted by smoking status (never/former/current).

c Further adjusted by urinary creatinine.

**Table 3:** Odds ratio (95% CIs) of PAD comparing the 75th with the 25th percentile of the metal distribution.

	Cases	Noncases	Model 1*^a^*	Model 2*^b^*	Model 3*^c^*
Cadmium	49	676	2.67 (1.40–5.07)	2.14 (1.11–4.13)	3.05 (0.97–9.58)
Lead	54	736	1.17 (0.81–1.69)	1.17 (0.78–1.76)	0.89 (0.45–1.78)
Barium	45	659	1.02 (0.67–1.56)	1.07 (0.72–1.58)	0.88 (0.57–1.36)
Cobalt	54	736	1.21 (0.65–2.23)	1.22 (0.67–2.20)	1.01 (0.33–3.14)
Cesium	54	736	1.08 (0.73–1.60)	1.19 (0.78–1.80)	0.91 (0.33–2.48)
Molybdenum	49	679	0.98 (0.60–1.60)	1.10 (0.69–1.77)	0.83 (0.49–1.41)
Antimony	49	676	1.25 (0.93–1.68)	1.30 (0.95–1.77)	1.15 (0.81–1.63)
Thallium	54	722	0.96 (0.53–1.73)	1.18 (0.60–2.32)	0.87 (0.30–2.52)
Tungsten	51	700	2.45 (1.12–5.37)	2.23 (1.03–4.82)	2.25 (0.97–5.24)

a Adjusted by age, sex, race, and education.

b Further adjusted by smoking status (never/former/current).

c Further adjusted by urinary creatinine.
